# Study of Formal Thought disorder symptoms in adults with Level 1 Autism Spectrum Disorder: Correlations between TALD, ADOS-2, and other neuropsychological dimensions

**DOI:** 10.1192/j.eurpsy.2025.1865

**Published:** 2025-08-26

**Authors:** C. Ricci, F. Iannotta, A. Catino, M. S. Vescio, W. Del Monaco, C. Ceparano, A. de Bartolomeis, F. Iasevoli

**Affiliations:** 1 Section of Psychiatry, Department of Neuroscience, University of “Federico II”, Naples, Italy

## Abstract

**Introduction:**

While several studies have investigated language disorders in individuals with Autism Spectrum Disorder (ASD), few have specifically examined formal thought disorder (FTD) symptoms, particularly in adults.

**Objectives:**

This study aims to assess FTD symptoms in adults with Level 1 ASD using the Thought and Language Disorder (TALD) scale and to analyze correlations between TALD scores and other neuropsychological dimensions.

**Methods:**

The study included 23 adults with Level 1 ASD (16 males, 7 females). Inclusion criteria were: a diagnosis of Level 1 ASD and age between 18 and 50 years. Exclusion criteria included: presence of major psychiatric comorbidities, intellectual disability, and neurological disorders that could impair language abilities. A comprehensive evaulation was conducted using TIB, HAMD, PANSS, STAI-Y1 and Y2, TASIT (Parts 1, 2, and 3), Ekman Facial Expression Test, TAS-20, ADOS-2, RAADS, AQ, and EQ. Language and thought disorders were evaluated using the TALD scale. Statistical analyses were performed using SPSS software to examine correlations between TALD scores and other neuropsychological dimensions.

**Results:**

The study population had a mean age of 27.6 ± 8.22 years, comprising 16 men and 7 women. Total TALD scores showed no significant correlation with educational level (r = -0.028, p = 0.914). Results revealed a significant correlation between the total TALD score and the total TASIT-2 score (r = -0.679, p = 0.011). Additionally, the “manneristic speech” item correlated with paradoxical sarcasm on TASIT-2 (r = -0.620, p = 0.024) and with lie recognition on TASIT-3 (r = -0.633, p = 0.02). The total ADOS-2 score showed a strong correlation with the total TALD score (r = 0.607, p = 0.028) and with specific linguistic features, including verbigeration (r = 0.725, p = 0.005), pressured speech (r = 0.648, p = 0.017), clanging (r = 0.725, p = 0.005), echolalia (r = 0.725, p = 0.005), and concretism (r = 0.738, p = 0.004). Furthermore, TALD was predictive of higher ADOS-2 scores (β = 0.607, t = 2.53, r = 0.607, p = 0.028, R² = 0.368).

**Conclusions:**

Our findings highlight specific FTD symptoms in adults with Level 1 ASD. The TALD scale proved to be a valuable tool, showing significant correlations with other neuropsychological domains. TALD scale also demonstrated a reliable predictive ability for higher ADOS-2 scores and distinct linguistic characteristics associated with ASD. TALD scale is proposed as a useful tool in clinical evaluations, providing additional insights beyond traditional diagnostic tools and showing independence from educational level.

**Disclosure of Interest:**

None Declared

